# Keeping it trim: roles of neuraminidases in CNS function

**DOI:** 10.1007/s10719-018-9837-4

**Published:** 2018-08-07

**Authors:** Alexey V. Pshezhetsky, Mila Ashmarina

**Affiliations:** 1 Sainte-Justine Hospital Research Center, Department of Paediatrics, University of Montreal, CHU Ste-Justine, Centre de recherche, 3175 Côte-Sainte-Catherine, Montréal, Québec H3T 1C5 Canada; 2 Department of Anatomy and Cell Biology, McGill University, Montreal, H3A0C7 Canada

**Keywords:** Sialic acid, Polysialic acid, Lysosome, CNS, Ganglioside, Neuraminidase

## Abstract

The sialylated glyconjugates (SGC) are found in abundance on the surface of brain cells, where they form a dense array of glycans mediating cell/cell and cell/protein recognition in numerous physiological and pathological processes. Metabolic genetic blocks in processing and catabolism of SGC result in development of severe storage disorders, dominated by CNS involvement including marked neuroinflammation and neurodegeneration, the pathophysiological mechanisms of which are still discussed. SGC patterns in the brain are cell and organelle-specific, dynamic and maintained by highly coordinated processes of their biosynthesis, trafficking, processing and catabolism. The changes in the composition of SGC during development and aging of the brain cannot be explained based solely on the regulation of the SGC-synthesizing enzymes, sialyltransferases, suggesting that neuraminidases (sialidases) hydrolysing the removal of terminal sialic acid residues also play an essential role. In the current review we summarize the roles of three mammalian neuraminidases: neuraminidase 1, neuraminidase 3 and neuraminidase 4 in processing brain SGC. Emerging data demonstrate that these enzymes with different, yet overlapping expression patterns, intracellular localization and substrate specificity play essential roles in the physiology of the CNS.

## Sialoglycoconjugates in mammalian brain

Sialoglycoconjugates (SGC) are biological macromolecules containing sialic acids (Sia) as part of their glycan chains usually at the terminal position. SGC are abundant on the surface as well as on the inner side of lysosomal and endosomal membranes in all mammalian cells, forming a dense array of complex sialylated glycans [1]. Due to the diverse physical and chemical properties of Sia (more than 50 types are described, distinguished by the presence of different side chains), SGC are ideally suited to serve as recognition markers for molecular and cellular interactions during cell signalling, proliferation, differentiation, interaction, migration, and adhesion (reviewed in [2–4]). The content of SGC strongly depends on the cell and tissue type, and changes during development [5].

In the central nervous system (CNS), two types of SGC, gangliosides and proteins containing polysialic acid (PSA) play determining roles. The gangliosides carry ~65% of all sialic acids in the mammalian brain and their levels in the brain are ~10-fold higher as compared to other organs. They are especially enriched in the membranes of neurons, usually as part of distinct lipid patches or rafts, and have several important functions essential for myelination, neuritogenesis, synaptic plasticity, and transmission of nervous impulses (reviewed in [6]). They associate laterally with membrane proteins, including receptors and ion channels, to modulate their activities. Ganglioside glycans extend into the extracellular space and interact with glycan-binding proteins to mediate cell-protein and cell-cell interactions. The brain ganglioside composition has a high complexity, but four related species: GM1a, GD1a, GD1b and GT1b dominate the adult mammalian brain. Each of them plays specific role in the brain function. For example, GM1a regulates Na^+^ channels, and in combination with GD1a, neuronal Ca^2+^ homeostasis and maintains neuronal viability, conductivity and excitability [7, 8]. Besides as described in details further down, GM1a plays an essential role in the multifascet mechanism for neurite outgrowth. By interaction with myelin-associated glycoprotein (MAG) GD1a and GT1b, mediate axon-myelin interactions and help axonal protection, regeneration and stabilization [9, 10]. In the developing brain dominant GD3 ganglioside plays a crucial role in maintaining the self-renewal capacity of neural stem cells, and neurogenesis by sustaining epidermal growth factor receptor (EGFR) signalling [11]. Not surprizingly, gene-targeted mice devoid of complex GM1a, GD1a, GD1b, GT1b showed weight loss, progressive motor and sensory dysfunction, and memory defects [12]. Similarly, mice with a deficiency in GD3 ganglioside and its derivatives had impaired brain neurogenesis in the hippocampus resulting from a progressive loss in neural stem cell population, and exhibited depression-like behavior [13].

4,8-Linked PSA chains are attached to a number of brain glycoproteins, the major beeng the Neural cell adhesion molecule (NCAM) and Synaptic cell adhesion molecule (SynCAM). On the neurons PSA play major roles by appropriately spacing the cells and shielding their surface receptors and ligands. Changes in PSA content have been implicated in the control of cell-cell and cell-matrix interactions, essential for appropriate brain development, adult brain plasticity, and nerve regeneration (reviewed in [6]). Dysregulation of PSA synthesis has been associated with multiple psychiatric disorders, including schizophrenia, bipolar disorder, and autism spectrum disorders [14, 15]. Besides NCAM and SynCAM, PSA have been identified on at least two brain microglia glycoproteins, Neurophilin 2 (NRP2) and E-selectin 1 (ESL-1). PSA are released upon activation of microglia and presumably play a role in negative feedback regulation reducing release of pro-inflammatory cytokines [16, 17].

In mammals, the synthesis of SGC is performed by a family of 20 sialyltransferases (ST) that catalyze the transfer of Sia from CMP-Sia to an acceptor carbohydrate [18]. Inactivating mutations in genes of the ST family have been linked to several human neurological disorders and behaviour abnormalities in the mouse strains, providing important clues about the function of SGC in the brain. In particular the deficiency of ST3GAL5 (GM3 synthase) causes Amish infantile epilepsy syndrome (OMIM 609056), an autosomal recessive syndrome associated with developmental stagnation and blindness [19]. Defects in the ST3GAL3 gene encoding β-galactoside-alpha-2,3-sialyltransferase-III cause autosomal recessive nonsyndromic mental retardation-12 (MRT12; OMIM 611090) [20] and early infantile epileptic encephalopathy-15 (EIEE15; OMIM 615006) [21]. In mouse models the loss of *St8sia2* resulted in decreased social interaction associated with synaptic defects [22, 23] while genetic inactivation of *St 3 gal4* caused a bleeding disorder associated with autosomal recessive thrombocytopenia due to abnormal sialylation of von Willebrand factor [24].

## Neuraminidases 1, 3 and 4 are the main SGC processing enzymes in the brain

The enzymatic removal of Sia is mediated by neuraminidases (sialidases) that are present in the majority of vertebrates as well as in a variety of microorganisms. Four types of neuraminidases (NEU) designated as NEU1, NEU2, NEU3 and NEU4 are present in mammalians (reviewed in [25, 26]). These enzymes have different, yet overlapping substrate specificity, tissue expression and intracellular localization. NEU1 is active primarily against sialylated glycopeptides and oligosaccharides with negligible activity against gangliosides. NEU3 requires a hydrophobic aglycone [27], which makes it active mainly towards gangliosides [28] whereas NEU4 is active against all types of sialylated glycoconjugates including oligosaccharides, glycoproteins and gangliosides [29]. Neuraminidases also show different specificity at the level of glycan structures: all neuraminidases show high activity against α2,3-linked Sia [25], while α2,6-linked Sia are readily cleaved by NEU3 and NEU1 but not by NEU4. The presence of a branching 2-Fuc inhibits Neu4, but has almost no effect on NEU1 or NEU3. The nature of the sugar residue at the reducing end, either glucose (Glc) or N-acetyl-D-glucosamine (GlcNAc), only has a minor effect on all neuraminidases, whereas core structure, 1,3 or 1,4 bond between D-galactose (Gal) and GlcNAc, was found to be important for Neu4 strongly preferring the β3 (core 1) to the β4 (core 2) isomer [30]. Importantly, neuraminidases also cleave α2,8 linkages. NEU3 hydrolyses α2,8 and α2,3-Sia with the similar rate but NEU1 and NEU2 show preference for α2,3-Sia [25]. Both NEU1 and NEU4 are able to cleave 4,8-linked Sia polymers [31].

NEU1 is ubiquitously expressed with the highest levels in the kidney, pancreas, skeletal muscle, liver, lungs, placenta and brain [32]. In these tissues, NEU1 generally shows 10–20 times higher expression than NEU3 and NEU4, and ~10^3^–10^2^ higher expression than NEU2 [33]. NEU2 is generally expressed at lower levels with an exception for the placenta and testis [34]. NEU3 has the highest expression in the adrenal gland, skeletal muscle, heart, testis and thymus [35, 36], while NEU4 is highly expressed in the brain, skeletal muscle, heart, placenta and liver [29, 33, 37].

In the mouse brain, NEU1, NEU3 and NEU4 show similar expression, each enzyme contributing to ~30% of total acidic neuraminidase activity against the synthetic substrate, 2′-(4-methylumbelliferyl)-α-D-N-acetylneuraminic acid (4MU-NeuAc). In the brain tissues of *Neu1, Neu3* or *Neu4* knockout (KO) mice, the activity is reduced to 70% of that in the wild-type (WT) brain, while in the brain of *Neu3/Neu4* double-knockout mice, the residual activity does not exceed 30% of the WT [27, 38]. Similar values were obtained by measuring neuraminidase activity in the presence of specific inhibitors of individual neuraminidase enzymes [39].

Importantly, while all 3 enzymes are expressed in the CNS neurons, they show unique expression patterns. *Neu1* is expressed ubiquitously throughout the brain but more strongly in specific areas such as the CA3 region of the hippocampus and the cortex while *Neu3* expression does not show a specific pattern [40]. In mice, NEU3 mRNA and protein levels in the hippocampus are high at early developmental stages (E15) and low in adulthood [41]. In contrast *Neu4* expression in the mouse or rat brain is undetectable at the embryonic stage, but rapidly increases at 3–14 days after birth [42, 43] when the enzyme is expressed in neurons scattered throughout the brain with a greater density in the hippocampus and in the ventral cortex [40, 44]. The exact identity of these neurons still awaits investigation. Besides, a recent study suggested the existence of mechanisms for compensatory regulation between the *Neu3* and *Neu4* genes [38] since the levels of *Neu3* and *Neu4* mRNA were significantly increased in the brain tissues of *Neu4* and *Neu3* single KO mice, respectively [38].

In the cell, NEU3 is localized in the caveolae microdomains of plasma, and endosomal membranes, i.e. compartments associated with the endocytic/recycling pathway [45, 46]. The enzyme was also detected on the surface of exosomes [47]. NEU3 was extracted from the membranes by sodium carbonate suggesting that it is a peripheral membrane protein [47], however a recent report provided certain evidence that NEU3 is an S-acylated transmembrane protein with the C-terminus exposed to the cytosol and the rest of the protein to the extracellular space [48]. NEU1 is localized at the lysosomal and plasma membranes [49, 50]. Similarly to NEU3 the nature of its interaction with the membrane is the subject of debate. Lukong *et al*. have demonstrated that NEU1 is an integral membrane protein with a C-terminal transmembrane domain and a cytosolic lysosomal targeting signal [49]. They also proposed that in the lysosome, the transmembrane domain of NEU1 can be further cleaved, resulting in a soluble form of the enzyme that has been purified from several mammalian tissues (reviewed in [51]). In contrast, structural models of NEU1 (reviewed in [26]) do not predict the existence of trans-membrane segments, suggesting that NEU1 rather associates with the membrane by lipid anchors, or binds to a membrane protein. A recent report, however, proposed that NEU1 contains two novel potential transmembrane domains with one of them also involved in the dimerization of the enzyme [52]. The *NEU4* gene is spliced into two forms differing in the first 12 N-terminal amino acids [33, 53]. The short isoform was found predominantly associated with ER membranes [33, 53], whereas the long form was reported to be present in lysosomes [29] and mitochondria [33, 54]. Because the substrate specificities of NEU1, NEU3 and NEU4 partially overlap, their distinct cellular and subcellular distribution may be the key to understanding the physiological functions of these enzymes. In contrast to other mammalian, bacterial or viral neuraminidases, NEU1 is activated by association with the lysosomal serine carboxypeptidase cathepsin A (protective protein, CTSA). CTSA, NEU1, and lysosomal β-galactosidase (GLB1) form a lysosomal multienzyme complex, where CTSA activates NEU1 and protects NEU1 and GLB1 against proteolytic degradation by lysosomal peptidases (reviewed in [51]).

## NEU1: Lysosomal catabolism, missfolded proteins and neurodegenerative diseases

Genetic deficiency of NEU1 in humans results in a severe metabolic disease, sialidosis (OMIM #256550), caused by the lysosomal storage of sialylated glycoproteins and oligosaccharides (reviewed in [55]). In addition, the genetic deficiency of CTSA results in the secondary deficiencies of NEU1 and GLB1, and causes the lysosomal storage disorder (LSD) galactosialidosis (GS OMIM #256540) (reviewed in [56]). Both disorders clinically manifest with skeletal and gait abnormalities, progressive impaired vision, bilateral macular cherry-red spots, ataxia, seizures and myoclonus syndrome. Severe early-onset forms are also associated with a dysmorphic phenotype, dysostosis multiplex, mental retardation and hepatosplenomegaly [57, 58]. A recent MRI study involving 11 attenuated sialidosis patients described increased mean diffusivity throughout the brain together with compromised white matter tracts integrity, the most severely affected cortical region being the occipital lobe. They also detected decreased functional connectivity from the temporal and occipital lobes to the hippocampus and parahippocampus and diffused cortical atrophy with posterior focal lesions potentially related to cortical blindness due to an altered neural network and a compromised visual pathway in the patients [59]. However, due to the rarity of the disease (1:4,000,000 live births [60]) and unavailability of CNS materials from human patients, the majority of the insights into the pathological CNS changes in sialidosis and GS came from studying the mouse models of the diseases. KO mouse models of sialidosis (*Neu1* KO) and galactosialidosis (*Ctsa* KO) develop phenotypes closely resembling severe systemic disease in human patients [57, 58]. Interestingly, the hypomorph *Ctsa* mouse with secondary 90% deficiency of NEU1 does not develop sialidosis suggesting that 10% of residual enzymatic activity is sufficient to support the required rate of lysosomal SGC catabolism [61]. The first reports on both sialidosis and GS mice described extensive brain pathology with lysosomal storage primarily observed in microglia and perivascular macrophages and to lesser extent in neurons. Neurodegeneration involving primarily Purkinje cells in the cerebellum was increased in GS as compared with sialidosis mice, which the authors attributed to the deficiency of *Ctsa* activity in the GS mouse [57, 58]. However, this hypothesis has not been further confirmed in the *Ctsa* knock-in model expressing an enzymatically mutant CTSA protein with the inactivating S190A mutation in the active site [61].

**Fig. 1:**
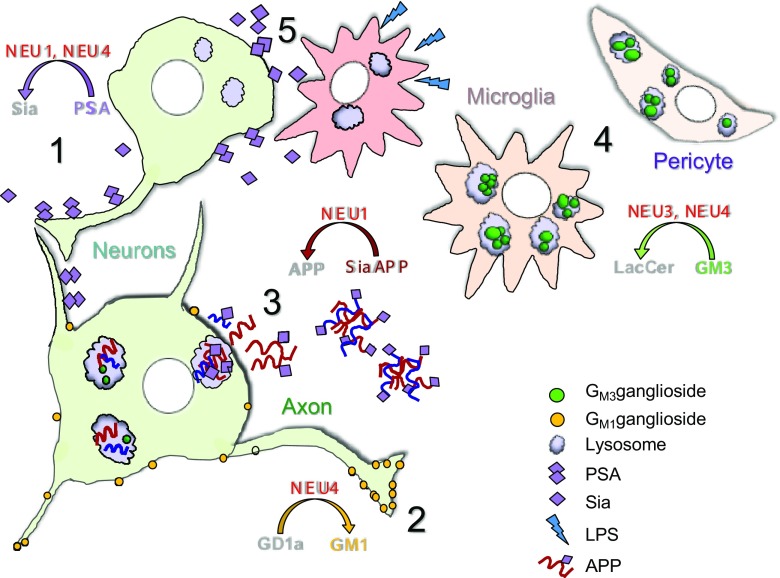
Proposed roles of neuraminidases in the CNS. 1. NEU1 and/or NEU4 reduce PSA content on neuronal surface during neuronal proliferation, differentiation, migration as well as during long-term potentiation of synapses. **2.** NEU4 generates GM1 ganglioside from GD1a ganglioside at the tip of the axon and induces its growth. **3.** NEU1 catalyzes lysosomal degradation of sialylated glycoproteins including APP. In sialidosis in the absence of Neu1, oversialylated APP is abnormally processed in lysosomes and the generated Aβ–peptide is released in the extracellular space. **4.** NEU3 and NEU4 convert GM3 to LacCer in the lysosomes. In the Neu3/Neu4 double knockout mice, GM3 is stored in microglia, vascular pericytes and neurons, causing micro- and astrogliosis, neuroinflammation, and accumulation of lipofuscin bodies. **5.** During neuroinflammation overexpressed NEU1 desialylates glycoproteins on cortical neurons

Further studies of neurodegeneration in the *Neu1* KO mice revealed the presence of amyloid bodies primarily clustered in the CA3 region of the hippocampus and resembling Alzheimer β-amyloid plaques [62]. The majority of these bodies were also immunoreactive for ubiquitin and neurofilaments. The authors showed that the amyloid precursor protein is a substrate of NEU1 and is oversialylated and accumulated in the lysosomes in the brain tissues of *Neu1* KO mice, perhaps due to a reduced catabolic rate. They further detected increased extracellular release of Aβ peptides by excessive lysosomal exocytosis, previously attributed to increased levels of the lysosomal membrane protein 1 (LAMP-1) showing elevated sialylation and a longer half-life in the lysosome in the absence of NEU1 [63]. Moreover, inactivation of the *Neu1* gene in the transgenic 5XFAD mice overexpressing human mutant amyloid precursor protein (APP) augmented formation of the plaques whereas intracerebral injections of adeno-associated viruses (AAVs) expressing NEU1 and CTSA could slow down or revert the amyloidogenic process, suggesting that reduced NEU1 brain levels could represent a risk factor for developing Alzheimer disease in humans. It is important to note, however, that the formation of cerebral amyloid plaques, lipofuscine granules and pTau aggregates has been reported for several mouse models of LSDs including MPS IIIA, B and C, MPS I and others, and has been attributed to autophagy block and impaired catabolism of missfolded proteins (see for example [64] or [65]) (Fig. 1). Therefore, it remains to be investigated to what extent the *Neu1* KO mice show augmented amyloidogenesis as compared with other neurological LSDs.

It also have been proposed that NEU1 is involved in the degradation of another missfolded pathogenic brain protein, prion (PrP^Sc^). PrP^Sc^ is a conformationally altered, self-replicating state of the prion protein PrP^C^. Importantly, PrP^Sc^ is sialylated by sialyltransferases of the St6Gal family and this modification plays a major role in evading innate immunity and infecting a host [66]. Significantly higher amounts of the C1 fragment relative to full-length PrP^C^ were detected in the brains of *Neu1* KO mice as compared to WT, *Neu3* or *Neu4* KO mice, suggesting that catabolism of PrP^C^ involves a desialylation of its proteolytic C1 fragments by NEU1 and consequent fast degradation of the desialylated products [67]. However, further studies are necessary to understand if NEU1 plays any role in predisposition to prion disease.

## Regulation of brain polysialic acids: NEU4 or NEU1?

PSA levels on the brain cells undergo rapid changes both during development and in the adulthood and neuraminidases together with metalloproteases have been proposed to play a role in this process. For example, rapid negative changes in neuraminidase activity in response to treatment with cortical steroids or chronic stress were detected in rat hippocampus and linked to increased PSA levels suggesting the existence of a pathway for regulation of synaptic plasticity by a brain neuraminidase(s) [68]. Takahashi [69] *et al*. demonstrated that the long form of murine NEU4 could hydrolyze oligoSia and PSA chains *in vitro* at a much higher rate than NEU1, NEU2 or NEU3, and could also remove PSA from NCAM when added to mouse brain tissue homogenates. Overexpression of NEU4 in ST8SiaIV-transfected neuroblastoma Neuro2a cells decreased PSA levels whereas silencing the endogenous *Neu4* in mouse embryonic hippocampal primary neurons increased both the PSA levels on neurites and their outgrowth. In contrast, transfection of the neurons with a NEU4-encoding plasmid reduced both the number of neurites and their length, allowing the authors to suggest that the enzyme is involved in both PSA degradation and regulation of neuronal differentiation [69]. Indirectly this conclusion was also supported by the author’s previous work demonstrateing that the expression of NEU4 in the mouse brain hippocampus peaked at post-natal days 3–14 coinciding with a massive reduction of PSA in the brain [43].

It is important to note, however, that because of the overlapping specificity of neuraminidases (for example NEU1. NEU2 and NEU4 show a comparable capacity to degrade 2,8-linked Neu5Ac polymers [31]), the results obtained by silencing or overexpressing individual isoforms in cellulo cannot be considered as an ultimate proof of their physiological function. Moreover, further work suggested that NEU1 but not NEU4 catabolizes PSA and trims it off brain cell surface glycoproteins. In particular, NEU1 has been implicated in stripping PSA from the surface of hippocampal granule cells (GCs) and is essential for proper migration of these neurons into the innermost region of the granule cell layer (GCL) of the hippocampus [70]. NEU1 knockdown in newly generated GCs *in vivo* both increased PSA presence on these cells and attenuated proper termination of GC migration in the innermost GCL suggesting that NEU1-mediated PSA removal is essential for proper neuronal lamination [70]. Sumida *et a*l. [71] reported that NEU1 also cleaves PSA on brain microglial cells. They demonstrated that NEU1 is rapidly released as a part of exovesicles by microglial cell line Ra2 upon an inflammatory stimulus. The secreted NEU1 clears PSA on the Ra2 surface and triggers the release of brain-derived neurotrophic factor retained by PSA molecules [71]. Most recently, Demina *et al*. (submitted) demonstrated that NEU1 is involved in the reduction of PSA on neuronal proteins following postnatal lipopolysaccharide (LPS) exposure in rat pups (a model of brain infections in preterm born children). Following intracranial injection of LPS, they detected a significant increase of the acidic neuraminidase activity and *Neu1* mRNA in the upper cortical layers. This was accompanied by a long-term reduction of sialylation of N-glycans on brain glycoproteins and reduced PSA levels on the surface of neurons in the upper somatosensory cortex. The authors hypothesize that neonatal LPS exposure results in specific and sustained induction of NEU1, causing long-lasting negative changes in sialylation and polysialylation of glycoproteins on brain cells and potentially contributing to pathophysiological consequences of perinatal infectious exposure (Fig. 1).

## Catabolism of brain gangliosides: NEU1, NEU3 or NEU4?

Metabolic genetic blocks in processing and catabolism of gangliosides result in the development of severe disorders, gangliosidoses, such as GM1 gangliosidosis, Tay-Sachs and Sandhoff diseases, which are all dominated by CNS involvement with marked neuroinflammation and neurodegeneration (reviewed in [72]). The ganglioside degradation pathway involves several desialylation steps (i.e. conversion of GD1a to GM1a, GM3 to lactosyl ceramide (LacCer) etc.) presumably catalyzed by the enzymes of the NEU family. However, no gangliosidoses caused by genetic deficiencies of human neuraminidases have been identified so far. Of all mammalian neuraminidases, NEU3 and NEU4 have the highest *in vitro* activity against gangliosides (reviewed in [25, 26, 73]) and are major candidates for being ganglioside catabolizing enzymes. In support of this, GM3 accumulation has been observed in a NEU3-silenced human glioblastoma cell line [74]. However, the *Neu4* and *Neu3* KO mouse models do not develop massive lysosomal storage of any ganglioside or severe clinical phenotypes resembling those of the *Glb1* KO mouse (model of GM1 gangliosidosis) or β-hexosaminidase deficient *Hexb* KO mouse (model of Sandhoff disease) [40, 75]. The *Neu3/Neu4* double-KO mice, however, store GM3 in the lysosomes of the brain’s phagocytic cells, microglia and pericytes, which gain a foamy appearance, accumulate multivesicular bodies similar to those present in GM1 and GM2 gangliosidoses, get activated, and secret inflammatory cytokines [38]. While brain phagocytic cells stored the majority of GM3 gangliosides, they are also accumulated in neurons, resulting in appearance of lysosomes containing whorls of membranes as well as enlarged lipofuscin bodies comprising multivesicular structures [38]. *In vitro*, neuraminidase activity against GM3 has been reduced to below detection level. However, GM3 increase in the brains of *Neu3/Neu4* double-KO mice was relatively modest as compared with those caused by deficiencies of the essential ganglioside hydrolases such as β-galactosidase (G_M1_ gangliosidosis) or β-hexosaminidases A and B (G_M2_ gangliosidosis), suggesting the existence of alternative catabolic pathways.

One of such pathways exists in the mouse model of Tay-Sachs disease (*Hexa*^*−/−*^ mouse), where the stored GM2 ganglioside is converted to GA2, then further hydrolyzed by β-hexosaminidase B to LacCer [76]. The pathway does not exist in humans, presumably because of lower levels of brain ganglioside neuraminidase activity. Three neuraminidases (NEU1, NEU3 and NEU4) have been proposed to be responsible for desialylation of GM2 in the mouse brain. First, Igdoura *et al*., showed a reduction of GM2 in the neuroglia cell line from a Tay-Sachs patient overexpressing human NEU1 [77]. They speculated that pharmacological induction or activation of this enzyme could be exploited for the treatment of Tay-Sachs disease [77]. Further studies, however, demonstrated that NEU1 has negligible activity against ganglioside substrates, in contrast with NEU3 and NEU4 [29]. In agreement with this, *Hexa*^*−/-*^*CathA*^*S190A-neo*^ mice with secondary 90% deficiency of NEU1 in the tissues do not show an increase in brain GM2 levels as compared with *Hexa*^*−/−*^ mice [78]. Li 2001 *et al*. tested if recombinant NEU2 and NEU3 could desialylate GM1 and GM2 gangliosides *in vitro* and found that the mouse NEU3 but not NEU2 was able to convert GM1 and GM2 into their respective asialo-derivatives in the presence of human or mouse GM2 activator protein [79]. Seyrantepe *et al*. further demonstrated that transfection with a NEU4-expressing plasmid can also rescue GM2 metabolism in the Tay-Sachs neuroglial cells [44]. However, double *Neu4/Hexa* KO mice showed only partially aggravated GM2 storage and clinical phenotype still milder than the Sandhoff mouse model [44]. These data suggested that NEU4 is not the only sialidase contributing to the metabolic bypass in *Hexa*^*−/−*^ mice. The question of which neuraminidase catabolizes GM2 was answered when Pan *et al*. [38] demonstrated that NEU3 deficiency in *Hexa*^*−/−*^ mice resulted in a vast (at least 5-fold) increase of stored brain GM2, suggesting that NEU3 is the enzyme responsible for the metabolic bypass. Independently, Seyrantepe *et al*. [80] conducted a thorough phenotypic characterization of double-knockout *Hexa*^*−/-*^*Neu3*^*−/−*^ mice and determined that, in contrast with *Hexa*^*−/−*^ mice that had a late onset at 10–11 months, the double-KO mice showed rapid deterioration with slow movement, ataxia and tremors and had a life span of only 1.5 to 4.5 months, similar to that of the Sandhoff *Hexb*^*−/−*^ mice. The *Hexa*^*−/-*^*Neu3*^*−/−*^ mice also had progressive neurodegeneration and astrogliosis mimicking the neuropathological and clinical abnormalities of the early-onset Tay-Sachs patients [80].

Potential role of the long NEU4 isoform (NEU4L) in the degradation of the b-series ganglioside GD3 is of special interest. GD3 is highly expressed in mouse and human embryonic neural stem cells but it becomes a minor component of the mature brain (reviewed in [6]). Hasegawa *et al*. published an observation that *Neu4* RNA and protein levels are rapidly decreasing during the apoptosis of neuroblastoma cells induced by the overexpression of tyrosinase that generated toxic catechol metabolites [81]. This process was concomitant with the increase of the mitochondrial pool of GD3, presumably degraded by NEU4L. At the same time, the authors did not present evidence that the increase of GD3 was the consequence of the NEU4 decrease or if accumulation of mitochondrial GD3 was involved in the neuronal apoptosis induced by catechol metabolites.

## Generation of GM1 ganglioside and induction of axonal growth: NEU3 or NEU4?

Ganglioside content and composition of the mammalian brain constantly change over an individual’s lifetime. Early in development (such as E14 in the rat), the mammalian brain is dominated by GM3 and GD3 gangliosides [82, 83]. By the time of birth, GM3 and GD3 become minor components in the brain, with GD1a and GT1b taking their place. Finally, in the mature brain, GM1a, GD1a, GD1b, and GT1b are present at comparable levels, together representing ~97% of gangliosides [84].

Until now, it has been assumed that the brain ganglioside composition is mainly regulated by changes in the rate of their biosynthesis in the Golgi, catalyzed by glycosyltransferases including sialyltransferases (reviewed in [6]). Genetic deficiencies of individual glycosyltransferases in human patients or in gene-targeted mouse models result in both changes in ganglioside profiles and severe neurological manifestations (reviewed in [6]). These mice lack the whole series of gangliosides: GD1b and GT1b in *St8sia1* knockout (KO) mice, GM1, GD1a, GD1b, and GT1b in *B4galnt1* KO mice or GD1a and GT1b in *St 3 gal2/3*-double KO mice [85, 86].

At the same time, multiple studies provide evidence that neuronal GM1 ganglioside is also generated by neuraminidases and that this process is essential for axonal growth and regeneration. During peripheral axon growth, GM1a binds to laminin-1 and induces its focal aggregation, in turn enhancing the relocation of tropomyosin receptor kinase A (TrkA) in lipid rafts and the subsequent activation of downstream signaling molecules [87]. Clustering of GM1 with laminin-1, along with laminin-1 self-assembly, also promotes relocation and enrichment of β1-integrin in lipid rafts and enhances combined laminin-integrin signaling to trigger neurite outgrowth [87]. Galectin-1 (Gal-1) was recently reported to cross-link GM1 ganglioside and α5 β1-integrin [88]. In normal cerebellar granule neurons and neuroblastoma NG108–15 cells, this led to a transient increase in intracellular Ca^2+^ by opening TRPC5 channels whereas cells from mice lacking major gangliosides, including GM1, showed retarded axonogenesis, underscoring the importance of GM1 for this process [88]. Conversion of GD1a to GM1 also reduces affinity for myelin-associated glycoprotein (MAG), identified as a ganglioside binding partner participating in in axon-myelin interactions [89]. Ganglioside-MAG interactions stabilize the axonal myelination, but they can also inhibit axon outgrowth and regeneration (reviewed in [6]). Moreover, in some types of nerve cells, MAG inhibition of axon outgrowth was reversed by cleaving the terminal sialic acids from GD1a and GT1b by exogenous sialidase (reviewed in [6]).

Several studies argued that NEU3 is the enzyme generating GM1 gangliosides at the growing axonal tip. First, Rodriguez *et al*. demonstrated that treatment of cultured differentiating hippocampal embryonic mouse neurons with pan-neuraminidase inhibitor, 2,3-dehydro-2-deoxy-N-acetylneuraminic acid (DANA) blocks axonal elongation, whereas transfection of the cells with NEU3 dramatically enhances axon growth and accelerates the polarization of cytoskeletal proteins [41]. Overexpression also resulted in the regrowth of the original axon but not of other neurites [41]. Proshin *et al*. reported that in human neuroblastoma NB-1 cells, expression of NEU3 was increased by dibutyryl cAMP which also induced neurite outgrowth [90]. Moreover, NB-1 cells transfected with a NEU3-expressing plasmid demonstrated more prominent outgrowth of neurites with axon-like characteristics, even in the absence of dibutyryl cAMP. Finally Da Silva *et al*. deciphered the mechanism by which NEU3 may induce axonal growth [91]. They demonstrated that NEU3 (referred to in the paper as plasma membrane ganglioside sialidase, PMGS) asymmetrically accumulates at the tip of one neurite of the unpolarized rat neuron, inducing actin instability. Suppression of NEU3 expression by siRNA and inhibition of neuraminidase activity by DANA blocked axonal generation, whereas neurons overexpressing a hemagglutinin (HA)-NEU3 fusion protein showed enhanced local TrkA activity, which further triggered phosphatidylinositol-3-kinase (PI3K)- and Rac1-dependent inhibition of RhoA signaling and actin depolymerization in the growing axon [91]. HA-NEU3 and TrkA could be co-immunoprecipitated from the neuronal membrane extracts, suggesting that they interact in the cell. The authors also claimed that pTrkA and HA-NEU3 interacted with GM1, because they could coprecipitate both proteins using cholera toxin B-modified beads. However, this experiment needs to be taken with a grain of salt since recent studies show that, besides GM1 ganglioside, cholera toxin also interacts with a number of gangliosides and even fucosylated glycoproteins [92].

Surprisingly, Valaperta *et al*. [93] reported that shRNA reduction of NEU3 in Neuro2a murine neuroblastoma cells did not inhibit axonal growth, but in contrast caused neurite elongation and appearance of the axonal marker protein Tau [93]. In NEU3 silenced cells, both GM1 and GD1a were decreased while GM2 increased, which was inconsistent with the hypothesis about the generation of GM1 from GD1a by NEU3 [93]. Most recently, Kappagantula *et al*. confirmed that NEU3 generates GM1 during the regeneneration of axons in the peripheral nerves but not of retinal axons in the CNS [94]. In peripheral axons of adult rats, NEU3 was activated and GM1/GD1a and GM1/GT1b ratios increased whereas no change in GM1/GD1a ratio or NEU3 expression was observed in retinal axons after axotomy, despite the presence of NEU3 in these cells. Finally, Woronowicz *et al*. proposed that TrkA is directly activated by a neuronal neuraminidase that cleaves a specific sialyl alpha-2,3-linked beta-galactosyl sugar residue on the receptor [95]. They reported that PC12 cells transfected with a TrkA-expressing plasmid as well as primary cortical neurons showed an increase of plasma neuraminidase activity in response to brain-derived neurotrophic factor (BDNF) [95]. Both the increase in neuraminidase activity and neurite outgrowth could be suppressed by an inhibitor of influenza virus neuraminidase, Tamiflu (Zanamavir), but not by DANA which, according to the authors, is specific towards NEU2 and NEU3 [95]. Other reports however have shown that DANA equally inhibits all 4 mamalian neuraminidases (IC50 between 10 and 50 μM), while Zanamavir shows the highest specificity towards NEU3 (IC50 4 μM), is less active against NEU2 and NEU4 (IC50 8 and 50 μM, respectively) and hardly shows any activity against NEU1 (IC50 > 500 μM) [27, 96–98]. Therefore, it is hard to conclude based on the reported data the activation of which NEU isoenzyme has been detected by the authors.

Importantly, as in the case of PSA removal described above, the main proof for the NEU3’s role in GM1 generation/axonal growth has been obtained *in vitro* either by treating embryonic neuronal cultures with unspecific neuraminidase inhibitor DANA or by overexpressing NEU3, which leaves open the question whether this also happens in the brain, where neuronal differentiation occurs in the presence of different cell types expressing different neuraminidases. This question has been partially addressed by the study of Pan [38] *et al*. in which they detected reduced GM1 in proximity to myelin layers in hippocampal and cortical neurons from both *Neu4* KO and double *Neu3/Neu4* KO mice. About 50% reduced GM1 and 30% increased GD1a levels were detected in total brains from both strains as compared with the WT levels. GM1a gangliosides were also reduced at the growing tip of the axons in cultured cortical neurons of the *Neu3/Neu4* double-KO mice (Fig. 1). Besides, NEU3/NEU4 combined deficiency significantly decreased neuritogenesis *in vitro* and the density of MAP2-positive neurofilaments *in vivo*. The authors conclude that the simplest explanation for low neuronal levels of GM1 ganglioside is that it is produced from GD1a by the action of NEU4 while admitting that further analysis of the ganglioside metabolism is necessary to prove this point [38].

## Rapid changes in cerebral neuraminidase activity associated with memory consolidation and seizures: NEU1?

Since the level of sialic acids on the cell surface as well as inside the cell changes during long-term potentiation (LTP), brain neuraminidase(s) were suggested to participate in neuronal plasticity. Initial evidences for this involvement were obtained by treating acute rodent hippocampal slices with pan-neuraminidase inhibitor DANA. In particular, Isaeva *et al*. demonstrated that DANA treatment increased the firing frequency and amplitude of spontaneous synchronous oscillations, the frequency of multiple unit activity and the tonic phase of seizure-like activity in the low-magnesium model of ictogenesis [99]. Pretreatment of hippocampal cultured brain slices with DANA also increased the density of simple and perforated synapses in the CA1 stratum radiatum region. Interestingly, high concentrations of calcium eliminated the effect of DANA on synaptic density, suggesting that synaptogenesis observed in response to neuraminidase treatment is an activity-dependent process [99].

More recently, the development of sensitive fluorogenic neuraminidase substrates and methods of live imaging allowed to investigate whether such processes occur *in vivo*. Minami *et al*. developed a highly sensitive histochemical imaging probe for sialidase activity, BTP3-Neu5Ac, which is cleaved by all 4 neuraminidases and demonstrated that, at neutral pH, the probe stained mossy fiber terminal fields in rat hippocampal acute slices [100]. The authors further showed that neuraminidase inhibitor DANA significantly impaired LTP at mossy fiber-CA3 pyramidal cell synapse. In rats, DANA injection into the hippocampal CA3 region of NEU4 knockdown rats prolonged the escape latency time in Morris water maze test suggesting that the regulation of sialylation by NEU4 is involved in hippocampal memory processing [100]. The same team further reported that neuraminidase activity at the CA3 stratum of rat acute hippocampal slices, visualized with BTP3-Neu5Ac, was rapidly increased in response to LTP-inducible high-frequency stimulation. This was accompanied by a rapid increase in free Sia levels, presumably shed from the neuronal surface [101]. Neuronal desialylation also occurred in live animals during hippocampus-dependent memory formation in a contextual fear-conditioning paradigm [101].

Interestingly, rapid changes in neuraminidase activity (decrease in cerebral cortex and cerebellum and increase in brainstem, hypothalamus and hippocampus) were also reported for the brains of WT kindled epileptic and non-epileptic seizing rats [102]. The authors speculate that these changes in activity may reflect functional and anatomical plastic changes associated with seizures [102]. Although providing a potentially interesting observation, the study does not allow to conclude which neuraminidases were responsible for the above changes.

## Conclusion

Multiple data endorse the important biological roles played by mammalian neuraminidases in the development and functioning of the CNS providing a rationale for identifying the main biological targets of neuraminidases in the brain. The availability of mouse models and recent development of high-throughput methods for analysis of sialoglycoconjugates through their metabolic labeling, advanced mass-spectrocopy and live imaging deliver a unique opportunity to compare the sialome (total complements of sialylated glycoproteins) of cells, tissues or organs from WT and KO mice. These animal models also provide a key tool for functional studies to link biochemical deficits of neuraminidases to biological phenotypes, which can only be defined by using the living organism. In addition, recent advances in the development of selective inhibitors of human neuraminidases [39, 97] provide researchers with pharmacological methods to target these enzymes *in vitro* and *in vivo*.

NEU1 deficiency causes severe systemic disorders, sialidosis and GS associated with pathological changes in the CNS. Although monogenic disorders caused by deficiencies of other neuraminidases are not described, it is possible that changes in sialylation can cause effects strong enough to influence predisposition to common neurological diseases (such as Alzheimer, Parkinson or ASD) but not strong enough to cause a Mendelian disease, therefore contributing to “hidden heritability” in human populations. Further studies are necessary to test this hypothesis by linking changes in neuraminidase activity and cell sialylation to pathological phenotypes.
